# First Data on WGS-Based Typing and Antimicrobial Resistance of Human *Salmonella* Enteritidis Isolates in Greece

**DOI:** 10.3390/antibiotics13080708

**Published:** 2024-07-29

**Authors:** Michalis Polemis, Theologia Sideroglou, Anthi Chrysostomou, Georgia D. Mandilara

**Affiliations:** 1 Computational Biology Department, Central Public Health Laboratory, National Public Health Organization (EODY), 16672 Vari, Greece; m.polemis@eody.gov.gr; 2 Directorate of Epidemiological Surveillance and Intervention for Infectious Diseases, National Public Health Organization (EODY), 15123 Maroussi, Greece; t.sideroglou@eody.gov.gr (T.S.); a.chrysostomou@eody.gov.gr (A.C.); 3 National Reference Centre for Salmonella, Faculty of Public Health Policies, School of Public Health, University of West Attica, 11521 Athens, Greece

**Keywords:** *S.* Enteritidis, surveillance, whole-genome sequence-based typing, phylogenetic analysis

## Abstract

*Salmonella enterica* subsp. *enterica* serotype Enteritidis (*S.* Enteritidis) is one of the major causes of foodborne infections and is responsible for many national and multi-country foodborne outbreaks worldwide. In Greece, human salmonellosis is a mandatory notifiable disease, with laboratory surveillance being on a voluntary basis. This study aims to provide the first insights into the genetic characteristics and antimicrobial resistance profiles of 47 *S.* Enteritidis human isolates using whole-genome sequencing (WGS) technology. The *S.* Enteritidis population was mainly resistant to fluoroquinolones due to *gyrA* point mutations, whereas one isolate presented a multi-resistant plasmid-mediated phenotype. ST11 was the most frequent sequence type, and phylogenetic analysis through the cgMLST and SNP methods revealed considerable genetic diversity. Regarding virulence factors, 8 out of the 24 known SPIs and C63PI were detected. Due to the observed variability between countries, it is of utmost importance to record the circulating *S.* Enteritidis strains’ structure and genomic epidemiology at the national level. WGS is a valuable tool that is revolutionizing our approach to *Salmonella* by providing a deeper understanding of these pathogens and their impact on human health.

## 1. Introduction

Non-typhoidal *Salmonella* continues, in the twenty-first century, to be a significant foodborne pathogen worldwide for humans [1]. *Salmonella* is prevalent in animals such as poultry, pigs, cattle, and pets. The bacterium is omnipresent and hardy, able to survive for long periods in a dry environment and several months in water [2,3]. Salmonellosis in humans is generally contracted through the consumption of contaminated food of animal origin, mainly eggs and poultry, but also from undercooked meat, unpasteurized dairy products, and fresh produce and fruits [4]. Symptoms include abdominal cramps, bloody diarrhea, fever, myalgia, headache, nausea, and vomiting [5]. *Salmonella* is globally estimated to cause 93 million enteric infections and 155,000 diarrheal deaths each year [6]. In European Union (EU) countries, salmonellosis is the second most common zoonotic disease after campylobacteriosis, and it is a major cause of foodborne disease outbreaks [4,7,8]. In 2022, there were 65,208 confirmed cases of human salmonellosis, corresponding to an EU notification rate of 15.3 cases per 100,000 population; *Salmonella* was the causative agent of 1014 foodborne outbreaks [4].

Antimicrobial resistance (AMR) in food-producing animals poses a significant global risk to public health, and it is among the leading health challenges of the century [9,10]. AMR bacteria, including *Salmonella*, can be transmitted to humans mainly through the consumption or handling of contaminated animal-derived food products or through direct contact with animals [11]. The European Commission, in 2017, adopted an Action Plan to tackle AMR following the One-Health approach that addresses resistance in bacteria infecting both humans and animals. The Action Plan includes the monitoring and reporting of AMR in the EU [12]. *Salmonella* strains with AMR have been identified in animals, animal-derived food products, and humans [12,13].

*Salmonella enterica enterica* serotype Enteritidis (SE) and *Salmonella enterica enterica* serotype Typhimurium are the two most prominent serotypes of *Salmonella* associated with human infections in most parts of the world [14]. By the 1980s, SE had emerged as a major concern for food safety in Europe and the USA, and since then, it has been the most frequently reported *Salmonella* serotype [15,16]. *Salmonella* Enteritidis has been extensively studied worldwide due to its significant impact on public health. Previous studies have highlighted its genetic diversity, virulence factors, and mechanisms of antimicrobial resistance [15,17,18]. Food-producing animals, particularly poultry and livestock, and their products, mainly eggs, are regarded as the most common sources of SE infection in humans. It is believed that SE has found a biological niche in table eggs [18,19], contaminating them through horizontal transmission [20] but also colonizing the ovarian tissue of hens and contaminating eggs during their formation through vertical transmission, a fact that causes massive economic losses to the poultry industry and leads to infections in humans [21,22,23]. In EU countries, SE is the most common serotype in human salmonellosis; in 2022, it represented 54.6% of confirmed human cases, and it was primarily related to “broiler” sources and also to “layers and eggs” [13]. SE is very often implicated in nationwide and multi-country outbreaks, the majority of which have been related to the consumption of raw or undercooked eggs or egg-containing foods and chicken meat [24,25,26]. As far as AMR in human SE isolates is concerned, in EU countries, SE resistance to ciprofloxacin was found in 22.8% of human isolates in 2022; resistance to ampicillin, sulfonamides, or tetracycline was observed in lower percentages [12].

In Greece, human salmonellosis is a mandatory notifiable disease; the National Public Health Organization and local public health authorities are notified about newly diagnosed cases. In parallel, human *Salmonella* isolates are sent to the National Reference Centre for *Salmonella* (NRCS) for further typing. The laboratory surveillance system is voluntary, and all isolates routinely undergo serotyping and antimicrobial susceptibility testing. Until 2022, molecular typing was limited only to the Pulsed Field Gel Electrophoresis method and was performed either in outbreak investigations or in the context of certain epidemiological studies. In 2023, the NRCS was included in the FWD AMR-RefLabCap project (https://www.fwdamr-reflabcap.eu/about-fwd-amr-reflabcap, accessed on 20 June 2024), aiming mainly to support the surveillance of AMR in *Salmonella* in human samples in terms of the whole-genome sequence-based method. The World Health Organization promotes *Salmonella* whole-genome sequencing (WGS) surveillance for monitoring trends and performing multinational outbreak detection, evaluating and monitoring prevention and control programs, contributing to the assessment of the disease burden, and generating hypotheses on sources and transmission modes; the European Centre for Disease Prevention and Control (ECDC) supports and encourage harmonized WGS-based *Salmonella* surveillance across Europe [27,28].

In Greece, between 2004 and 2022, 11,665 salmonellosis cases were reported. The mean annual notification rate of salmonellosis was 5.6 cases per 100,000 population. SE was the most frequent serotype, accounting for almost 43% of serotyped human *Salmonella* isolates from 2018 to 2022 [29]. According to a previous study, human SE isolates from 2003 to 2020 were mainly resistant to fluoroquinolones (17.9%) and much less to penicillins (2.8%), cephalosporins (1.3%), aminoglycosides (1.4%), or tetracyclines (1%) [30].

This study aims to provide preliminary insights into the genetic characteristics and antimicrobial resistance profiles of SE isolated from clinical samples in Greece during 2023 using WGS technology. The research focuses on understanding the genetic diversity and resistance mechanisms of this pathogen.

## 2. Results

### 2.1. Plasmid Detection

Two plasmids were detected in 95.7% (45/47) of the SE isolates analyzed in this study, characterized as IncFIB(S) and IncFII(S). One of the isolates (ID 93/2023) (multi-drug-resistant—MDR, demonstrating resistance to kanamycin, tetracycline, and fluoroquinolones) carried two extra plasmids: IncI1-I(Alpha) and IncX1 (4 plasmids in total). Two isolates, both resistant to fluoroquinolones, carried no plasmids (Table 1).

### 2.2. Antimicrobial Resistance

Bioinformatic analysis of the sequenced, phenotypically (fluoro)quinolone-resistant SE isolates (32/47; 68.1%) revealed point mutations in the quinolone resistance-determining region (QRDR) of the chromosomal DNA gyrase gene: at codon 87, D87Y, *n* = 29, changing aspartate to tyrosine; D87N, *n* = 1, changing aspartate to asparagine; and at codon 83, S83Y, *n* = 1, changing serine to tyrosine. One SE isolate (ID 93/2023), phenotypically multi-resistant, was identified carrying the resistance genes *aph(3′)-la*, *qnrS13* (plasmid-mediated quinolone resistance—PMQR), and *tet(A)*, conferring resistance to kanamycin, ciprofloxacin, and tetracycline, respectively. All three genes were detected on the same contig carrying the plasmid IncI1-I(Alpha) (Table 1).

### 2.3. Genetic Diversity-cgMLST and SNP Analysis

**Figure 1:**
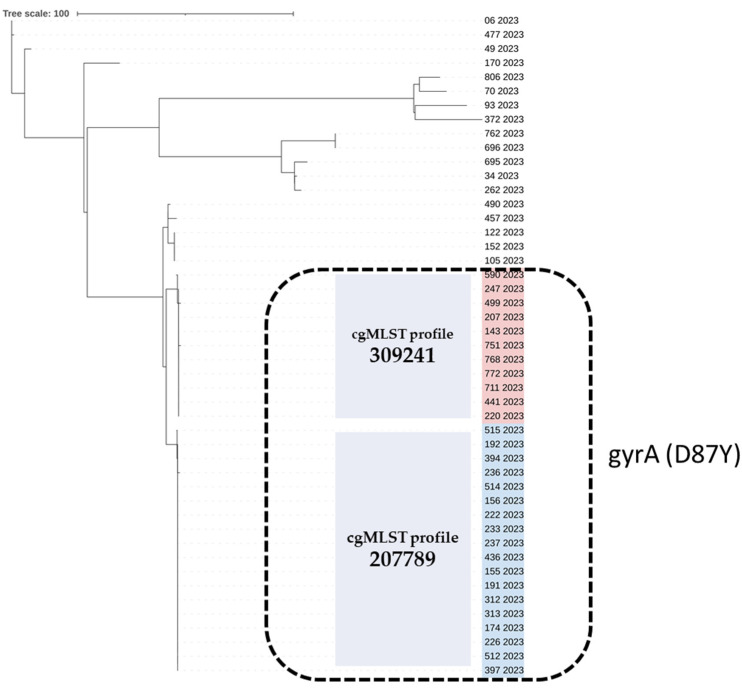
The cgMLST-based phylogenetic tree of the 47 *Salmonella* Enteritidis strains isolated from human samples in 2023, Greece. The strain ID and year of isolation are given for each strain. The two cgMLST profiles 309241 and 207789, comprising quinolone-resistant isolates carrying the *gyrA* (D87Y) point mutation (black, dashed line rectangle), are highlighted in red and blue, respectively. The neighbor-joining tree based on *S.* Enteritidis cgMLST allelic profiles determined by the chewBACCA software version 3.5 [31]. The dendrogram was visualized with iTOL [32].

All sequenced SE isolates belonged to Sequence Type 11 (ST11). However, core genome multilocus sequence typing (cgMLST) analysis revealed 14 different profiles among ST11 SE isolates. Eighteen of the fluoroquinolone-resistant isolates belonged to cgMLST profile 207789 (*n* = 18) and eleven to profile 309241 (*n* = 11). These two cgMLST profiles demonstrated high genetic similarity and included all isolates with the *gyrA* (D87Y) mutation. Isolates with *gyrA* D87N and S83Y mutations belonged to cgMLST 238619 and 61992, respectively. The multi-resistant SE isolate (ID 93/2023) (resistant to kanamycin, tetracycline, and fluoroquinolone) was cgMLST 251903. The 15 SE pan-susceptible isolates were resolved into nine different cgMLST profiles, each including 1 to 3 isolates (Table 1) (Figure 1).

**Figure 2:**
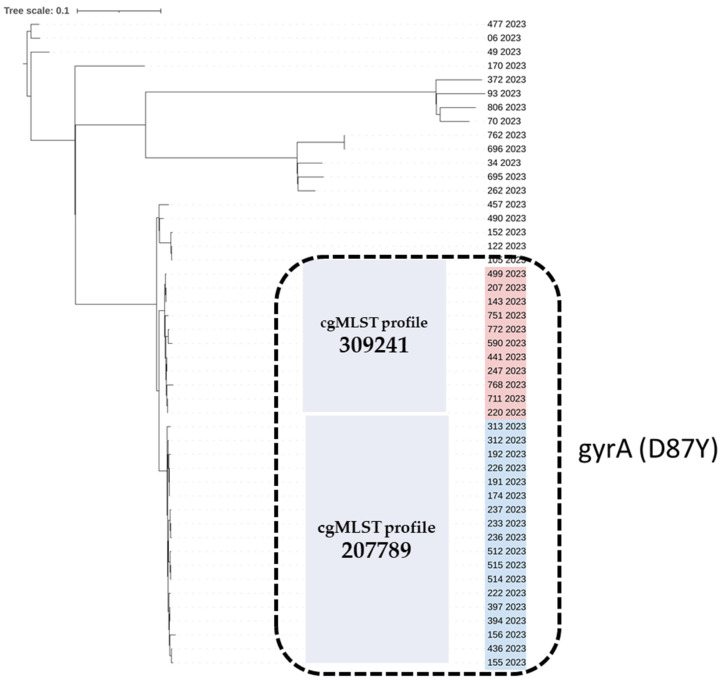
Single-Nucleotide Polymorphism (SNP)-based phylogenetic tree of the 47 *Salmonella* Enteritidis strains isolated from human samples in 2023, Greece. The strain ID and year of isolation are given for each strain. The quinolone-resistant *S.* Enteritidis isolates carrying the *gyrA* (D87Y) point mutation are marked with a black, dashed-line rectangle. The quinolone-resistant group of isolates comprises two cgMLST profiles, 309241 and 207789, which are highlighted in red and blue, respectively. SNP analysis was conducted using Snippy [33]; the maximum likelihood tree was constructed using RAxML [34] and visualized with iTOL [32].

Although Single-Nucleotide Polymorphism (SNP) analysis provided a higher level of resolution than cgMLST, the two derived trees have the same topology and lead to the same results; the fluoroquinolone-resistant isolates that include the *gyrA* (D87Y) mutation and belong to closely related cgMLST profiles 207789 (*n* = 18) and 309241 (*n* = 11) are arranged in a similar way in the two trees (Figure 1 and Figure 2).

### 2.4. Salmonella Pathogenicity Island (SPI) Identification

Regarding virulence factors, 8 out of the 24 known SPIs were detected in all isolates, including SPI-1, SPI-2, SPI-3, SPI-5, SPI-10, SPI-13, and SPI-14, as well as C63PI, and these were detected in all isolates. Some isolates also harbored SPI-4 (Table 1).

### 2.5. Assessment of the Genetic Relatedness of Greek Isolates in the Context of EnteroBase

**Figure 3:**
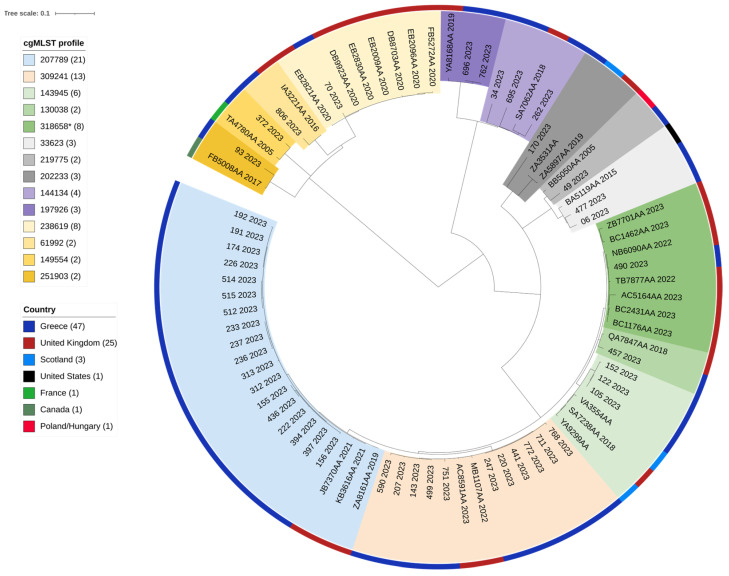
A Single-Nucleotide Polymorphism (SNP)-based phylogenetic tree of 79 *Salmonella* Enteritidis strains; 47 were isolated from human samples in 2023, Greece, and 32 were downloaded from Enterobase. The strain ID, cgMLST profile, originating country, and year of isolation are given for each strain. Both the cgMLST profile and originating country are highlighted with different colors, explained in the respective legends. SNP analysis was conducted using Snippy [33]; the maximum likelihood tree was constructed using RAxML [34] and visualized with iTOL [32]. * Of the 44 *S.* Enteritidis cgMLST profile 318658 genomes found in Enterobase (accessed on 16 July 2024), 7 representative isolates were included in the phylogenetic tree.

Of the Salmonella Enteritidis ST11 genomes deposited in Enterobase, submitted with a public release, 65 genomes from different countries were found to belong to the various cgMLST profiles identified in the Greek collection (Enterobase accessed on 16 July 2024). No Greek isolates were retrieved. Within each cluster, as determined by cgMLST, SNP analysis revealed that the isolates were closely related; in the two prevalent cgMLST profiles of the Greek genomes (cgMLST profile 207789 (*n* = 18) and profile 309241 (*n* = 11)), isolates from the United Kingdom were found to be closely grouped. Multi-resistant SE isolate 93/2023 with cgMLST profile 251903 was grouped with an SE specimen isolated in Canada in 2017 (Figure 3).

## 3. Discussion

*Salmonella* Enteritidis is responsible for many national and multi-country foodborne outbreaks. It is pivotal to recognize, in each country, the circulating strains’ structure and genomic epidemiology, since they vary between countries. In this study, we investigated 47 human isolates of *S.* Enteritidis in terms of genomic characterization and phylogenetic relationships.

The findings from this study provide a comprehensive genomic characterization of SE in Greece, highlighting significant genetic diversity and resistance patterns. The prevalence of ST11 among the isolates is consistent with previous reports from other European countries, indicating a widespread clonal lineage. This strain has been linked to foodborne illness outbreaks in recent years, particularly in Europe, and has been detected in various reservoirs, including humans, poultry, and food sources [25,26,35].

IncFIB(S) and IncFII(S) plasmids are genetic elements found in a wide variety of bacteria, including *Salmonella enterica* strains. They belong to the incompatibility group IncFIB and IncFII plasmids, respectively. They have been associated with carrying genes that confer resistance to various antibiotics. They can also harbor genes that enhance a bacterium’s virulence and are known to be readily transferred between bacteria through conjugation. This allows them to rapidly spread antibiotic resistance and virulence factors among different bacterial populations, posing a public health threat [17,36,37]. The present study revealed IncFIB(S) and IncFII(S) plasmids in all but two isolates, indicating that these plasmids have a widespread distribution in human SE isolates in Greece. However, antimicrobial genes were not found on the contigs carrying the IncFIB(S) and IncFII(S) sequence replicons.

One SE isolate (ID 93/2023) with multi-resistance to kanamycin, tetracycline, and fluoroquinolones harbored two extra plasmids, IncI1-I(Alpha) and IncX1. IncI1 plasmids are relatively common across various *Enterobacteriaceae* of food-animal origin and in human patients. They can carry genes for various purposes, including antibiotic resistance and virulence factors, e.g., genes that increase the severity of infections by enhancing bacterial adhesion or toxin production. IncI1 plasmids are a public health concern because they can contribute to the spread of antibiotic resistance among enteric bacteria [38,39]. The genes *aph(3′)-Ia*, *qnrS13*, and *tet(A)* of the MDR isolate ID 93/2023 that confer resistance to kanamycin, fluoroquinolones, and tetracycline, respectively, were found on the same contig carrying the IncI1-I(Alpha) plasmid. This carriage of multiple resistance genes is critical, especially due to the ability of IncI1 plasmids to be conjugally transferred among different bacteria. IncX1 plasmids are part of the IncX group of plasmids and have been found in *Enterobacteriaceae* members of various origins, including animal, environmental, and clinical sources. They play a significant role in the dissemination and stability of antibiotic resistance genes, with IncX1 subgroup plasmids exhibiting higher transfer rates than IncX2 plasmids [40]. However, antimicrobial genes were not found on the contig carrying the IncX1 sequence replicon of the ID 93/2023 isolate.

Quinolones are a class of broad-spectrum antibiotics used to treat a wide range of bacterial infections, and they exert their antibacterial effect by inhibiting bacterial DNA synthesis, targeting DNA gyrase and topoisomerase IV. Nalidixic acid and related first-generation antibiotics were only active against Gram-negative bacteria. Fluoroquinolones are the most common and effective subgroup of quinolones. They contain a fluorine atom in their chemical structure, making them more potent and broad-spectrum than basic quinolones [41]. Quinolones, particularly fluoroquinolones, have been used in various food-producing animals, including poultry, cattle, and swine, to treat and prevent bacterial infections [42,43]. There is strong evidence that the use of fluoroquinolones in animal production can contribute to the development of antibiotic-resistant bacteria. There is a high risk of transmitting these resistant strains to humans via the food chain, which makes infections difficult to treat [44,45]. Bacteria have developed various mechanisms to resist the effects of fluoroquinolone antibiotics: mutations that alter drug targets (amino acid substitutions in a region of the *GyrA* or *ParC* subunit, termed the “quinolone-resistance–determining region”—QRDR), mutations that reduce drug accumulation, and plasmids that protect cells from the lethal effects of quinolone (plasmid-mediated quinolone resistance—PMQR—through *qnr* genes that produce proteins that bind to and protect both DNA gyrase and topoisomerase IV from inhibition) [46]. The D87Y point mutation in the *gyrA* gene was detected in 61.7% (29/47) of the SE isolates; this point mutation alters the protein structure, affecting how fluoroquinolones bind, and it is considered a well-characterized mechanism of fluoroquinolone resistance in *Salmonella* Enteritidis. Other *gyrA* mutations, like S83Y and D87N, associated with highly resistant mutants were also detected, but at a low frequency (4.3%; 2/47) [25,47].

It is obvious that whole-genome sequencing (WGS) has revolutionized the field of microbial surveillance and characterization, including the detection of antimicrobial resistance (AMR) genes and the identification of plasmids harboring AMR genes in *Salmonella* and other pathogens.

The most commonly used WGS subtyping methods for the strain-level differentiation of *Salmonella* are the SNP and cgMLST methods. They have high discriminatory power and are invaluable in both epidemiological and outbreak investigations. For SNP analysis, single-nucleotide changes are used to determine phylogenetic relatedness between strains relative to a closely related reference sequence, whereas, for cgMLST, differences in the core genome loci of the isolates (loci found in at least 95–98% of the reference organism strains used to build the allele scheme) can be used to generate a phylogeny based on a subset of genes [48,49]. *Salmonella* Enteritidis, which is considered a highly clonal pathogen with limited genetic diversity, presents a challenge for WGS subtyping methods since, due to their high resolution, they can detect even subtle genetic differences between clonal isolates. An analysis of the sequences of the *n* = 47 ST11 *Salmonella* Enteritidis isolates in this study using both the SNP and cgMLST methods revealed considerable genetic diversity. The SNP analysis provided a greater resolution than cgMLST clusters; however, the two derived trees have the same topology and lead to the same results, indicating concordance between the two phylogenetic clustering analyses by the cgMLST and SNP approaches.

According to our results, several *Salmonella* pathogenicity islands (SPIs) were present in the *S.* Enteritidis genomes, including SPI-1, SPI-2, SPI-3, SPI-4, SPI-5, SPI-10, SPI-13, and SPI-14, as well as C63PI. *Salmonella* pathogenicity islands (SPIs) are clusters of genes within the *Salmonella* genome that encode virulence factors. These virulence factors are essential for causing disease in humans and animals. SPI-1 is crucial for initiating infection. It encodes a type III secretion system (T3SS) that acts like a molecular syringe. The T3SS injects bacterial proteins into host cells, facilitating the invasion and manipulation of the host environment. Once *Salmonella* invades host cells, SPI-2 becomes critical for its survival and replication within those cells. This SPI encodes genes for a capsule polysaccharide, a sugary coat that shields the bacteria from the host’s immune system. SPI-3 plays a role in intestinal inflammation during *Salmonella* infection. SPI-4 is involved in fimbrial adherence, and SPI-5 is involved in bacterial uptake of nutrients within the host. SPI-10, SPI-13, and SPI-14 are not extensively characterized in *Salmonella*. C63PI, also referred to as the centisome 63 pathogenicity island, is a region on the *Salmonella* chromosome that encodes genes involved in iron acquisition. Iron availability is limited within the host, and efficient iron acquisition systems, like those encoded by C63PI, are crucial for *Salmonella* to establish a successful infection [50].

The SNP analysis of the Greek *S.* Enteritidis strains and isolates from other countries (from a search of publicly available genomes) selected to be within the same cgMLST profiles resulted, in most cases, in closely related SNP clusters. Comparing whole-genome sequencing data of isolates from one country on a comprehensive online platform dedicated to the analysis and visualization of genomic variation, like Enterobase, is vital in terms of correlating strains and identifying international connections. Contributing data to resources for enteric bacterial genomics strengthens global disease surveillance and preparedness and allows for faster and more targeted public health interventions during outbreaks.

The results of our study are the first data on the WGS-based characterization of *Salmonella* Enteritidis isolates from clinical samples in Greece. Expanding the sample size will offer a more comprehensive understanding of *S.* Enteritidis epidemiology in Greece. It is obvious that whole-genome sequencing (WGS) is playing an increasingly important role in understanding, controlling, and preventing *Salmonella* infections. WGS-based typing methods provide a much more detailed genetic map of *Salmonella* isolates compared to traditional typing methods, contributing to the early identification of closely related clusters, potentially indicating outbreaks, thus allowing for the rapid identification and characterization of the strain involved and leading to quicker implementation of control measures to prevent further spread. Moreover, WGS data can be used to monitor the spread of specific *Salmonella* strains over time and across geographical regions. This can help public health officials identify emerging trends and potential threats. WGS can also identify genes associated with antibiotic resistance in *Salmonella* isolates. This allows for targeted surveillance of resistant strains and informs decisions regarding antibiotic use in animals and humans.

## 4. Materials and Methods

### 4.1. Sample Collection, Serotyping, and Antimicrobial Susceptibility Testing (AST)

In 2023, a total of 514 laboratory-confirmed *Salmonella* isolates were collected and serotyped by the National Reference Laboratory (NRL) for *Salmonella* in Greece. These isolates were derived from clinical samples, including stool, blood, and other bodily fluids. The serotyping of *Salmonella* spp. isolates was performed by the slide agglutination method according to the White–Kaufmann–Le Minor Scheme (Bio-rad Salmonella antisera, Hercules, CA, USA; Sifin Salmonella antisera, Berlin, Germany) [51]. Antimicrobial susceptibility testing was performed using the Kirby–Bauer disk diffusion method, following the European Committee on Antimicrobial Susceptibility Testing (EUCAST) guidelines [52]. The following antibiotics were tested (Oxoid™ Antimicrobial Susceptibility discs, Thermo Scientific, Waltham, MA, USA): (i) penicillins: ampicillin and amoxicillin–clavulanic acid; (ii) cephalosporins: ceftazidime, ceftriaxone, and cefotaxime; (iii) fluoroquinolones: ciprofloxacin, nalidixic acid, and pefloxacin; (iv) miscellaneous agents: chloramphenicol, trimethoprim, sulfamethoxazole-trimethoprim, and spectinomycin; (v) aminoglycosides: kanamycin, tobramycin, netilmicin, and streptomycin; (vi) carbapenems: meropenem; (vii) macrolides: azithromycin; and, finally, (viii) tetracyclines: tetracycline.

The predominant serotype was *S.* Enteritidis (273/514, 53.1%). AST performed for 191 *S.* Enteritidis isolates revealed resistance to two (fluoro)quinolones of the antibiotic panel, nalidixic acid and/or pefloxacin (129/191, 67.5%); one isolate was characterized as multi-resistant with additional resistance to kanamycin and tetracycline.

Whole-genome sequencing (WGS) was performed on 47 selected *S.* Enteritidis isolates identified in 2023. The criteria used for the selection of the isolates were based on geographical distribution (representing all 7 regions of the country), age, specimen, and antimicrobial resistance phenotype. Six human isolates from two different SE outbreaks that occurred in Greece in 2023 were also included (outbreak A, outbreak B). Table 1 illustrates the characteristics of the 47 SE isolates.

### 4.2. Whole-Genome Sequencing (WGS)

WGS was conducted on 47 *S.* Enteritidis isolates by Eurofins Genomics using the Illumina NovaSeq 6000 platform. High-quality genomic DNA was extracted and sequenced, resulting in high-coverage data for downstream analyses. The raw sequence reads were quality-checked, trimmed, and assembled into contigs.

### 4.3. Bioinformatic Analysis

The bioinformatics analysis was performed using the “*AMR” workflow (https://github.com/phac-nml/staramr, accessed on 10 June 2024), which incorporates several tools and databases, including MLST, ResFinder, PointFinder, and PlasmidFinder [53]. These tools facilitated the identification of sequence types (STs), resistance genes, point mutations, and plasmid content. Core genome multilocus sequence typing (cgMLST) was conducted using the Enterobase scheme (cgMLST V2 + HierCC V1), encompassing 3002 loci [54]. The cgMLST scheme data were downloaded on June 20, 2024. Single-Nucleotide Polymorphism (SNP) analysis was conducted using Snippy [33]; the maximum likelihood tree was constructed using RAxML [34] and visualized with iTOL [32]. Τhe identification of *Salmonella* pathogenicity islands (SPIs) was performed by submitting the complete nucleotide sequence to SPIFinder, available at the Center for Genomic Epidemiology web server (https://cge.cbs.dtu.dk/services/, accessed on 24 June 2024) (>95% Identity and >80% Coverage thresholds were used) [55]. A comparison of the studied *S.* Enteritidis isolates with related strains from Europe, the United States of America, and Canada was performed using isolates from Enterobase, which is considered the reference public genome database for Salmonella [56,57]. From the publicly available *Salmonella* Enteritidis ST11 genomes hosted in Enterobase, those assemblies found to belong to the cgMLST profiles identified in the Greek collection were retrieved, and the comparison was performed via SNP analysis and visualized, as described above.

## 5. Conclusions

This study presents the preliminary results of the WGS-based characterization of *S*. Enteritidis isolates from clinical samples in Greece. Our study provides essential baseline data that can inform public health strategies and interventions. However, the limited number of isolates sequenced and the focus on clinical samples represent limitations that should be addressed in future research. Expanding the sample size and including isolates from diverse sources, such as food and the environment, will offer a more comprehensive understanding of *S.* Enteritidis epidemiology in Greece.

## Figures and Tables

**Table 1:** Characteristics of 47 selected human *Salmonella* Enteritidis isolates, isolated in 2023 in Greece, based on WGS-based typing.

NRCS ID	Specimen	Age Group of Cases	AΜR Genotype	Sequence Type (ST)	cgMST Profile	Predicted AMR Phenotype	Plasmids	SPIs *
155	Stool	>65 years	*gyrA* (D87Y)	11	207789	ciprofloxacin, nalidixic acid	IncFIB(S), IncFII(S)	1,2,3,5,10,13,14 and C63PI
156	Stool	6–14 years	*gyrA* (D87Y)	11	207789	ciprofloxacin, nalidixic acid	None	1,2,3,4,5,10,13,14 and C63PI
174	Blood	15–64 years	*gyrA* (D87Y)	11	207789	ciprofloxacin, nalidixic acid	IncFIB(S), IncFII(S)	1,2,3,5,10,13,14 and C63PI
191	Stool	0–11 months	*gyrA* (D87Y)	11	207789	ciprofloxacin, nalidixic acid	IncFIB(S), IncFII(S)	1,2,3,4,5,10,13,14 and C63PI
192	Stool	0–11 months	*gyrA* (D87Y)	11	207789	ciprofloxacin, nalidixic acid	IncFIB(S), IncFII(S)	1,2,3,4,5,10,13,14 and C63PI
222	Stool	6–14 years	*gyrA* (D87Y)	11	207789	ciprofloxacin, nalidixic acid	IncFIB(S), IncFII(S)	1,2,3,5,10,13,14 and C63PI
226	Stool	1–5 years	*gyrA* (D87Y)	11	207789	ciprofloxacin, nalidixic acid	IncFIB(S), IncFII(S)	1,2,3,5,10,13,14 and C63PI
233 ** ^A^	Stool	1–5 years	*gyrA* (D87Y)	11	207789	ciprofloxacin, nalidixic acid	IncFIB(S), IncFII(S)	1,2,3,4,5,10,13,14 and C63PI
236 ** ^A^	Stool	15–64 years	*gyrA* (D87Y)	11	207789	ciprofloxacin, nalidixic acid	IncFIB(S), IncFII(S)	1,2,3,4,5,10,13,14 and C63PI
237 ** ^A^	Stool	15–64 years	*gyrA* (D87Y)	11	207789	ciprofloxacin, nalidixic acid	IncFIB(S), IncFII(S)	1,2,3,4,5,10,13,14 and C63PI
312	Stool	unknown	*gyrA* (D87Y)	11	207789	ciprofloxacin, nalidixic acid	IncFIB(S), IncFII(S)	1,2,3,5,10,13,14 and C63PI
313	Stool	unknown	*gyrA* (D87Y)	11	207789	ciprofloxacin, nalidixic acid	IncFIB(S), IncFII(S)	1,2,3,4,5,10,13,14 and C63PI
394	Stool	6–14 years	*gyrA* (D87Y)	11	207789	ciprofloxacin, nalidixic acid	IncFIB(S), IncFII(S)	1,2,3,5,10,13,14 and C63PI
397	Stool	15–64 years	*gyrA* (D87Y)	11	207789	ciprofloxacin, nalidixic acid	IncFIB(S), IncFII(S)	1,2,3,4,5,10,13,14 and C63PI
436	Stool	6–14 years	*gyrA* (D87Y)	11	207789	ciprofloxacin, nalidixic acid	IncFIB(S), IncFII(S)	1,2,3,5,10,13,14 and C63PI
512 ** ^B^	Stool	unknown	*gyrA* (D87Y)	11	207789	ciprofloxacin, nalidixic acid	IncFIB(S), IncFII(S)	1,2,3,4,5,10,13,14 and C63PI
514 ** ^B^	Stool	15–64 years	*gyrA* (D87Y)	11	207789	ciprofloxacin, nalidixic acid	IncFIB(S), IncFII(S)	1,2,3,4,5,10,13,14 and C63PI
515 ** ^B^	Stool	15–64 years	*gyrA* (D87Y)	11	207789	ciprofloxacin, nalidixic acid	IncFIB(S), IncFII(S)	1,2,3,4,5,10,13,14 and C63PI
143	Stool	1–5 years	*gyrA* (D87Y)	11	309241	ciprofloxacin, nalidixic acid	IncFIB(S), IncFII(S)	1,2,3,4,5,10,13,14 and C63PI
207	Stool	6–14 years	*gyrA* (D87Y)	11	309241	ciprofloxacin, nalidixic acid	IncFIB(S), IncFII(S)	1,2,3,4,5,10,13,14 and C63PI
220	Stool	6–14 years	*gyrA* (D87Y)	11	309241	ciprofloxacin, nalidixic acid	IncFIB(S), IncFII(S)	1,2,3,5,10,13,14 and C63PI
247	Stool	1–5 years	*gyrA* (D87Y)	11	309241	ciprofloxacin, nalidixic acid	IncFIB(S), IncFII(S)	1,2,3,5,10,13,14 and C63PI
441	Blood	>65 years	*gyrA* (D87Y)	11	309241	ciprofloxacin, nalidixic acid	IncFIB(S), IncFII(S)	1,2,3,5,10,13,14 and C63PI
499	Stool	15–64 years	*gyrA* (D87Y)	11	309241	ciprofloxacin, nalidixic acid	IncFIB(S), IncFII(S)	1,2,3,4,5,10,13,14 and C63PI
590	unknown	unknown	*gyrA* (D87Y)	11	309241	ciprofloxacin, nalidixic acid	IncFIB(S), IncFII(S)	1,2,3,4,5,10,13,14 and C63PI
711	Blood	unknown	*gyrA* (D87Y)	11	309241	ciprofloxacin, nalidixic acid	IncFIB(S), IncFII(S)	1,2,3,4,5,10,13,14 and C63PI
751	Blood	0–11 months	*gyrA* (D87Y)	11	309241	ciprofloxacin, nalidixic acid	IncFIB(S), IncFII(S)	1,2,3,5,10,13,14 and C63PI
768	Stool	1–5 years	*gyrA* (D87Y)	11	309241	ciprofloxacin, nalidixic acid	None	1,2,3,4,5,10,13,14 and C63PI
772	Stool	6–14 years	*gyrA* (D87Y)	11	309241	ciprofloxacin, nalidixic acid	IncFIB(S), IncFII(S)	1,2,3,5,10,13,14 and C63PI
457	Stool	unknown	None	11	130038	Susceptible	IncFIB(S), IncFII(S)	1,2,3,5,10,13,14 and C63PI
105	Stool	1–5 years	None	11	143945	Susceptible	IncFIB(S), IncFII(S)	1,2,3,4,5,10,13,14 and C63PI
122	Stool	6–14 years	None	11	143945	Susceptible	IncFIB(S), IncFII(S)	1,2,3,4,5,10,13,14 and C63PI
152	Stool	15–64 years	None	11	143945	Susceptible	IncFIB(S), IncFII(S)	1,2,3,4,5,10,13,14 and C63PI
490	Stool	6–14 years	None	11	318658	Susceptible	IncFIB(S), IncFII(S)	1,2,3,4,5,10,13,14 and C63PI
170	Stool	1–5 years	None	11	202233	Susceptible	IncFIB(S), IncFII(S)	1,2,3,5,10,13,14 and C63PI
6	Blood	>65 years	None	11	33623	Susceptible	IncFIB(S), IncFII(S)	1,2,3,4,5,10,13,14 and C63PI
477	Blood	unknown	None	11	33623	Susceptible	IncFIB(S), IncFII(S)	1,2,3,5,10,13,14 and C63PI
49	Blood	>65 years	None	11	219775	Susceptible	IncFIB(S), IncFII(S)	1,2,3,4,5,10,13,14 and C63PI
34	Blood	>65 years	None	11	144134	Susceptible	IncFIB(S), IncFII(S)	1,2,3,4,5,10,13,14 and C63PI
262	Stool	unknown	None	11	144134	Susceptible	IncFIB(S), IncFII(S)	1,2,3,5,10,13,14 and C63PI
695	Blood	>65 years	None	11	144134	Susceptible	IncFIB(S), IncFII(S)	1,2,3,5,10,13,14 and C63PI
696	Stool	6–14 years	None	11	197926	Susceptible	IncFIB(S), IncFII(S)	1,2,3,4,5,10,13,14 and C63PI
762	Stool	6–14 years	None	11	197926	Susceptible	IncFIB(S), IncFII(S)	1,2,3,5,10,13,14 and C63PI
372	Stool	unknown	None	11	149554	Susceptible	IncFIB(S), IncFII(S)	1,2,3,4,5,10,13,14 and C63PI
70	Stool	15–64 years	*gyrA* (D87N)	11	238619	ciprofloxacin, nalidixic acid	IncFIB(S), IncFII(S)	1,2,3,5,10,13,14 and C63PI
806	Blood	>65 years	*gyrA* (S83Y)	11	61992	ciprofloxacin, nalidixic acid	IncFIB(S), IncFII(S)	1,2,3,4,5,10,13,14 and C63PI
93	Stool	1–5 years	*aph*(3′)-*Ia*, *qnrS13*, *tet(A*)	11	251903	kanamycin, ciprofloxacin, tetracycline	IncFIB(S), IncFII(S), IncI1-I(Alpha), IncX1	1,2,3,4,5,10,13,14 and C63PI

* SPI-3, SPI-4, SPI-5, SPI-10, SPI-14, and C63PI were partially identified. ** *Salmonella* Enteritidis outbreak cases (^A^: outbreak A; ^B^: outbreak B) that occurred in Greece in 2023.

## Data Availability

The data presented in this study are available on request from the corresponding author. Data are uploaded on The European Surveillance System (TESSy) where access is granted only to authorized personnel.
